# Morphologic Changes in Circulating Blood Cells of COVID-19 Patients

**DOI:** 10.7759/cureus.13416

**Published:** 2021-02-18

**Authors:** Gagandeep Kaur, FNU Sandeep, Oluwaseyi Olayinka, Gunjan Gupta

**Affiliations:** 1 Pathology and Laboratory Medicine, Danbury Hospital, Danbury, USA

**Keywords:** covid-19, cbc, morphologic changes, blood cells

## Abstract

Objective

Since the beginning of the coronavirus disease 2019 (COVID-19) pandemic, many studies have described the quantitative peripheral blood findings seen in COVID-19 patients. However, morphologic changes have been described by only a few studies. We report morphologic and quantitative changes in peripheral blood of COVID-19 patients.

Design

We reviewed electronic medical records, complete blood counts, and peripheral blood smears of 20 patients who were COVID-19 positive by reverse transcriptase-polymerase chain reaction (RT-PCR), from March 1, 2020, through May 31, 2020. The peripheral blood smears of all 20 patients were retrieved and morphological features of white blood cells, red blood cells, and platelets were reviewed and documented. Appropriate pictures were taken.

Results

Of the 20 patients reviewed, 13 were males and seven were females. The average age of the patients was 65.1 years. The most common quantitative hematologic abnormalities noted on complete blood count (CBC) were anemia followed by neutrophilia, neutrophilic left shift, and lymphopenia. The most significant morphologic changes noted were neutrophils with clumped chromatin, multiple abnormal nuclear shapes, pseudo-Pelger-Huet deformity, and smudged neutrophils. Lymphocytes showed abundant blue cytoplasm and/or lymphoplasmacytoid morphology and monocytes were activated with abnormal shapes and vacuolization. Platelets were adequate in number in the majority of patients and platelet clumping was the most significant finding noted. The red blood cells were normocytic and normochromic with few nucleated red blood cells and coarse basophilic stippling.

Conclusion

Our study identifies and describes significant morphologic changes in the peripheral blood cells of COVID-19 patients. An understanding of these morphologic changes in addition to established hematologic parameters can aid in the diagnosis of COVID-19 and serial CBC and peripheral smear review may help with management decisions in COVID-19 patients.

## Introduction

Coronavirus disease 2019 (COVID-19) is caused by the new virus, severe acute respiratory syndrome coronavirus 2 (SARS-CoV-2), which was first identified in Wuhan, China in December 2019 [1]. As of February 10, 2021, a total of 107,300,544 cases have been confirmed globally with 2,352,098 global deaths [2]. Although the pathogenesis of COVID-19 is not fully understood, studies have shown that the virus which is transmitted primarily via respiratory droplets, penetrates the host cell by interacting with the angiotensin-converting enzyme 2 (ACE2), a monocarboxypeptidase present on the surface of many cell types including epithelial cells lining the respiratory tract and capillary endothelial cells [3]. Invasion of these cells by the virus activates a series of events that leads to derangement of the renin-angiotensin-aldosterone axis with consequent deleterious effects [4]. Activation of the immune system with subsequent immune dysregulation has also been implicated in the pathogenesis of COVID-19. Involvement of the immune system is evidenced by the presence of lymphopenia, particularly in those with severe disease [5]. While lymphopenia and other quantitative abnormalities in the peripheral blood have been well described in the literature, little is known about the morphologic changes in circulating blood cells in COVID-19 [6,7]. We aim to perform a retrospective study describing the morphologic abnormalities and identifying trends in the peripheral blood counts of COVID-19 patients. An understanding of these morphologic changes in addition to established hematologic parameters can aid in the diagnosis of COVID-19 and serial complete blood count (CBC) and peripheral smear review may help with management decisions in COVID-19 patients.

## Materials and methods

Hospital IRB approval was obtained prior to conducting this retrospective study. The laboratory information system was searched for COVID-19 patients who tested positive by reverse transcriptase-polymerase chain reaction (RT-PCR) from March 1, 2020, through May 31, 2020, and a peripheral blood smear slide prepared for review. The goal was to randomly identify and analyze data from at least 20 hospitalized patients. Upon identifying 20 patients meeting the above criteria, we searched their electronic medical records for the following information: age, sex, date of admission, admission into the intensive care unit (ICU), date of peripheral blood smear evaluation, date of discharge, and complete blood count including hemoglobin, hematocrit, mean corpuscular volume, platelet count, red blood cells, white blood cells and differentials, and reticulocyte count.

The peripheral blood smears of all 20 patients were retrieved and morphological features of white blood cells, red blood cells, and platelets were reviewed and documented. Appropriate pictures were taken.

## Results

Of the 20 patients reviewed, 13 were males and seven were females. The average age of the patients was 65.1 years. Thirteen patients were in the ICU at the time of peripheral blood smear review. The most common quantitative hematologic abnormalities noted were anemia and neutrophilia followed by lymphopenia (Table 1). 

**Table 1:** Study demographics and complete blood count findings ICU, intensive care unit; RBC, red blood cell; HB, hemoglobin; HCT, hematocrit; MCV, mean corpuscular volume; MCH, mean corpuscular hemoglobin; MCHC, mean corpuscular hemoglobin concentration; RDW-CV, red cell distribution width-coefficient of variation; WBC, white blood cell count; IG, immature granulocyte; F, female; M, male

Age	Sex	ICU	RBC (4.20–5.80) × 10(6)/uL	HB (13.5–17.0) g/dL	HCT (38.0–50.0) %	MCV (80–100) fL	MCH (25.0–34.0) pg	MCHC (31–36) g/dL	RDW CV (11–15) %	WBC (3.5–10.0) × 10(3)/uL	Absolute Neutrophil (2.00–7.50) × 10(9)/L	Absolute Lymphocyte (1.00–4.00) × 10(9)/L	Absolute Monocyte (0.00–1.00) × 10(9)/L	Absolute Eosinophil (0.00–0.50) × 10(9)/L	Absolute Basophil (0.00–0.20) × 10(9)/L	IG % Auto (0.0–1.0) %
58	F	Yes	3.02 L	9.4 L	29.7 L	98.3 N	31.1 N	31.6 N	16.2 H	4.4 N	3.26 N	0.84 L	0.18 N	0.04 N	0.00 N	1.4 H
71	M	Yes	5.00 N	13.1 L	41.9 N	83.8 N	26.2 N	31.3 N	22.9 H	18.9 H	17.58 H	0.38 L	0.76 N	0.00 N	0.00 N	3.2 H
71	M	Yes	4.90 N	14.5 N	41.8 N	85.3 N	29.6 N	34.7 N	13.7 N	1.0 L	0.29 L	0.46 L	0.23 N	0.02 N	0.00 N	0.00 N
81	F	Yes	4.77 N	13.3 L	43.0 N	90.1 N	27.9 N	30.9 L	15.5 H	6.4 N	3.65 N	1.76 N	0.63 N	0.21 N	0.06 N	0.3 N
55	M	Yes	2.57 L	8.5 L	26.1 L	101.6 H	33.1 N	32.6 N	17.3 H	8.7 N	7.70 H	0.48 L	0.27 N	0.10 N	0.01 N	0.7 N
72	M	Yes	4.00 L	12.7 L	35.7 L	89.3 N	31.8 N	35.6 N	14.6 N	13.5 H	12.83 H	0.27 L	0.41 N	0.00 N	0.00 N	0.9 N
71	F	Yes	4.79 N	12.7 N	43.1 N	90.0 N	26.5 N	29.5 L	17.4 H	23.0 H	15.08 H	5.85 H	1.45 H	0.01 N	0.10 N	2.0 H
77	M	Yes	3.42 L	9.7 L	30.7 L	89.8 N	28.4 N	31.6 N	15.5 H	22.3 H	19.07 H	0.77 L	1.17 H	0.39 N	0.07 N	1.9 H
32	M	Yes	2.31 L	6.6 L	19.9 L	86.0 N	28.6 N	33.2 N	15.5 H	16.3 H	9.78 H	3.75 N	0.33 N	0.82 H	0.16 N	2.6 H
74	M	Yes	2.21 L	6.7 L	22.0 L	95.6 N	30.1 N	30.5 L	15.7 H	52.1 H	41.68 H	2.61 N	4.17 H	0.00 N	0.00 N	4.5 H
54	M	Yes	3.07 L	7.7 L	24.8 L	80.8 N	25.1 N	29.8 L	15.5 H	39.2 H	34.24 H	2.76 N	3.29 H	0.00 N	0.24 H	19.5 H
62	F	Yes	2.81 L	8.1 L	23.6 L	83.9 N	28.4 N	33.6 N	15.9 H	10.2 H	7.51 H	1.17 N	0.31 N	0.03 N	0.01 N	4.3 H
77	M	Yes	3.26 L	9.8 L	29.9 L	91.7 N	30.1 N	32.5 N	17.2 H	7.9 N	6.79 N	0.63 L	0.32 N	0.00 N	0.00 N	0.9 N
53	F	No	4.37 N	13.5 N	41.1 N	94.1 N	30.9 N	32.8 N	13.1 N	5.5 N	4.02 N	0.99 L	0.44 N	0.00 N	0.00 N	0.5 N
89	M	No	2.90 L	8.6 L	25.6 L	88.3 N	29.7 N	33.6 N	16.7 H	11.6 H	10.79 H	0.35 L	0.35 N	0.00 N	0.00 N	5.1 H
88	M	No	4.15 L	13.6 N	41.8 N	100.7 H	32.8 N	32.5 N	14.6 N	5.9 N	5.05 N	0.57 L	0.26 N	0.00 N	0.00 N	0.8 N
60	M	No	4.10 L	13.0 L	38.0 N	92.7 N	31.7 N	34.2 N	12.1 N	4.5 N	3.78 N	0.27 L	0.27 N	0.00 N	0.00 N	0.6 N
33	F	No	2.14 L	7.5 L	22.0 L	102.8 H	35.0 H	34.1 N	19.3 H	14.0 H	11.36 H	1.22 N	1.07 H	0.23 N	0.03 N	0.8 N
68	M	No	3.06 L	9.2 L	28.2 L	92.9 N	30.1 N	32.6 N	12.7 N	26.4 H	21.0 H	2.78 N	1.34 H	0.00 N	0.04 N	4.6 H
56	F	No	3.89 N	8.0 L	27.5 L	70.7 L	20.6 L	29.1 L	16.9 H	9.7 N	8.63H	1.07 N	0.00 N	0.00 N	0.00 N	2.0 H

The morphologic changes that were noted in the peripheral blood cells upon microscopic examination are mentioned in the subsections below.

Neutrophils

**Figure 1:**
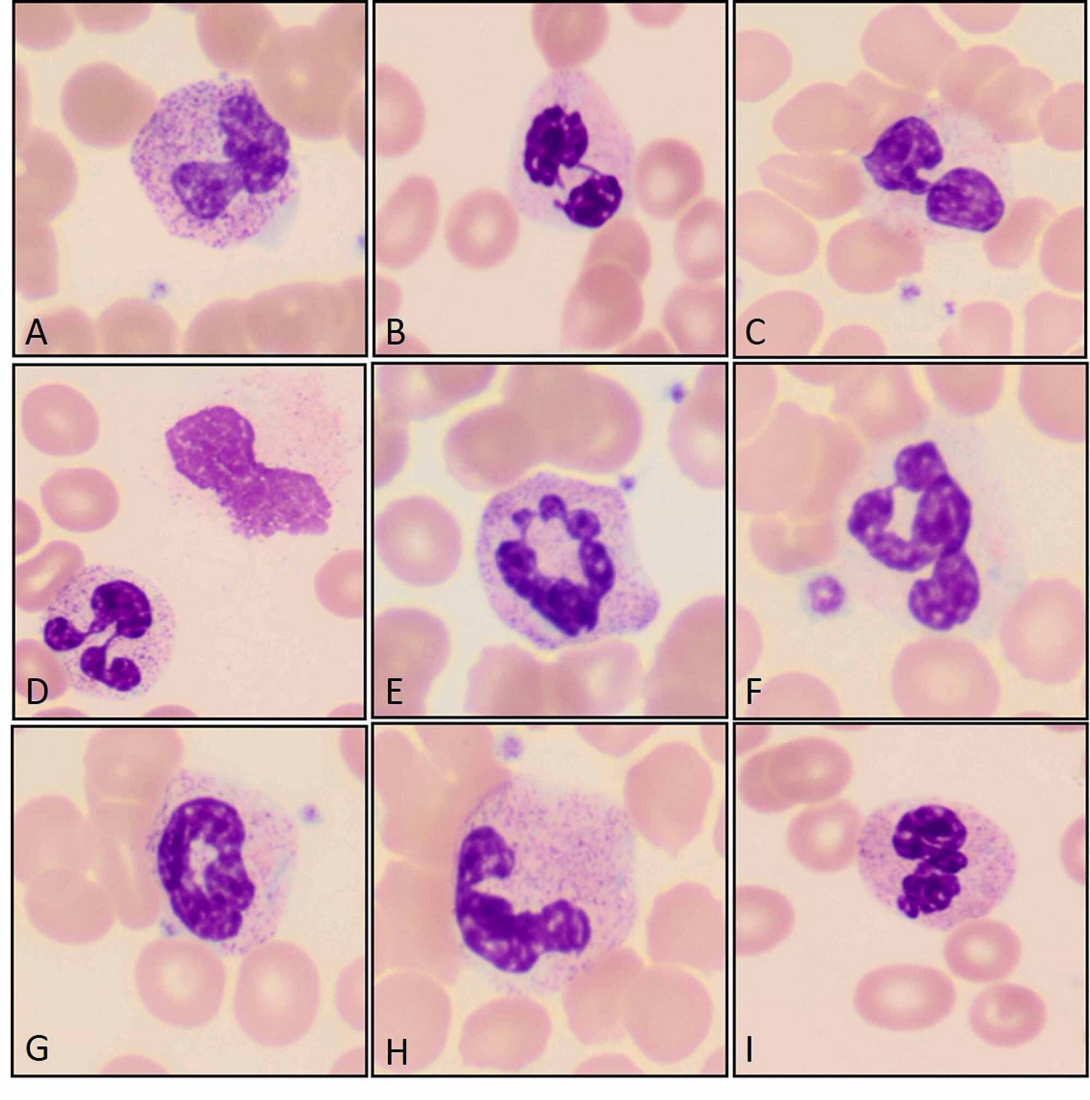
Left shifted neutrophils with clumped chromatin and toxic granulation (A), with pseudo-Pelger-Huet deformity (B and C), with pi-shaped nuclear deformity (D), and other abnormal shapes (E, F, and I) including ring neutrophils (G), and a fetus shaped nuclear deformity (H). (Original magnification ×1000 oil immersion {A through I}.)

Neutrophils showed clumped chromatin with toxic granulation and vacuolization. Multiple abnormal nuclear shapes including fetus-shaped nuclei, pi-shaped nuclei, C and donut-shaped nuclei as well as cells with aberrant nuclear projections were seen. The pseudo-Pelger-Huet deformity was also noted. One unique finding which has not been described in the literature was smudged neutrophils. This was found in most of the peripheral smears (Figures 1A-1I). In addition, neutrophilic left shift with bands, metamyelocytes, and myelocytes was observed in the smears of patients with neutrophilia.

Lymphocytes

**Figure 2:**
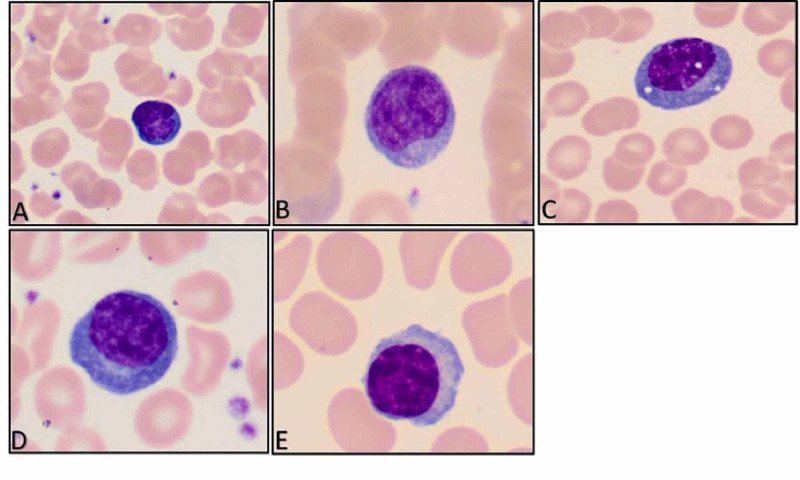
Lymphocytes with abundant pale to dark blue cytoplasm (A, B, and C), with lymphoplasmacytoid features (D and E). (Original magnification ×1000 oil immersion {A through E}.)

Microscopic examination showed increased numbers of large granular lymphocytes, lymphoplasmacytoid cells, and atypical lymphocytes with abundant blue cytoplasm (Figures 2A-2E). 

Monocytes

**Figure 3:**
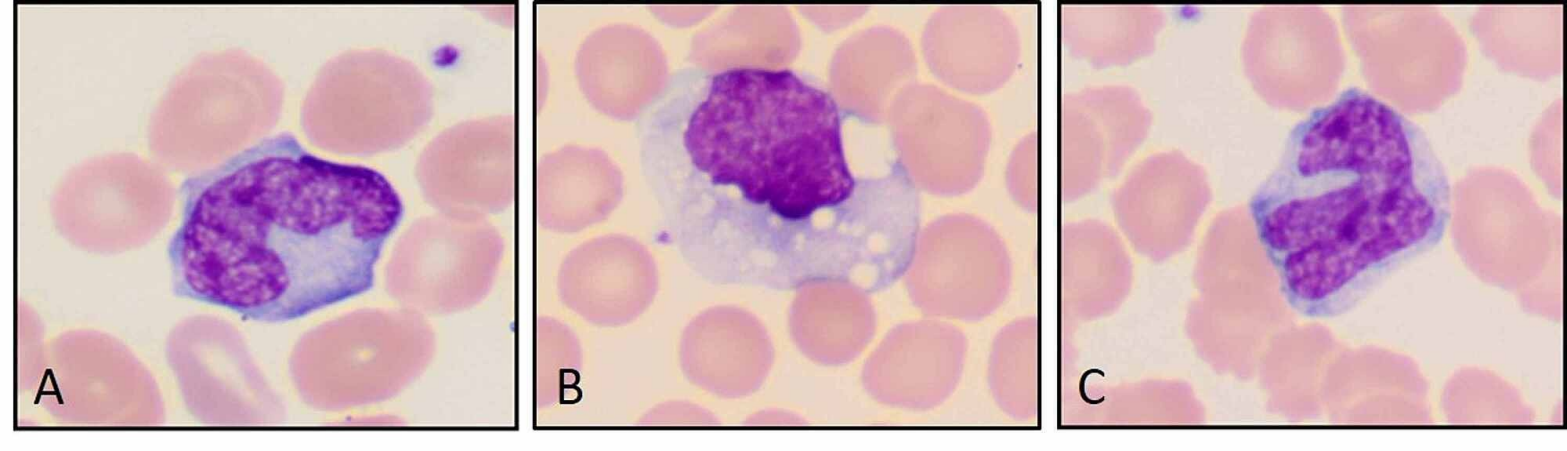
Activated macrophages with abnormal shapes and cytoplasmic vacuolization. (Original magnification ×1000 oil immersion {A through C}.)

Activated monocytes and monocytes with variable abnormal shapes and cytoplasmic vacuolization were seen (Figures 3A-3C).

Platelets

**Figure 4:**
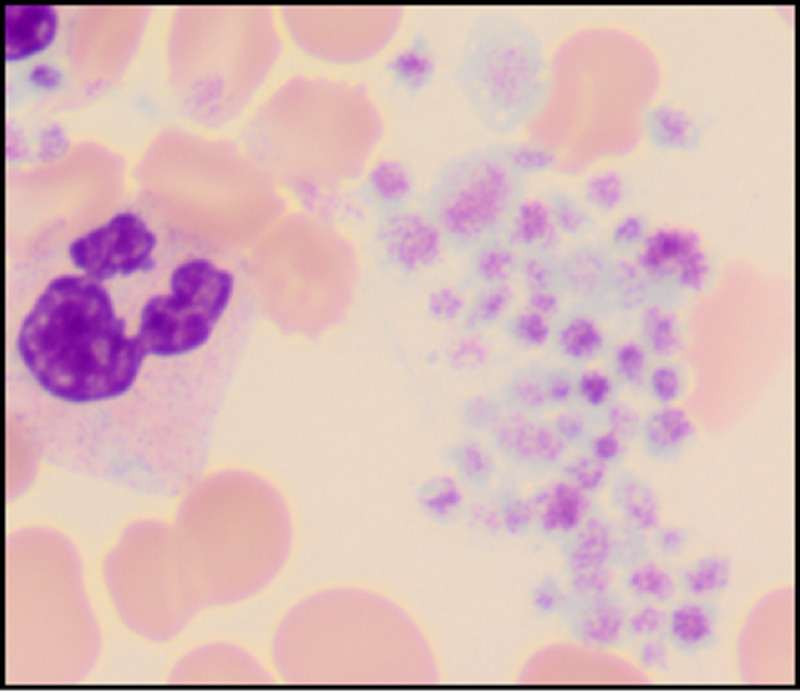
Platelet clumps. (Original magnification ×1000 oil immersion.)

Platelets were adequate in number in the majority of patients and platelet clumping was the most significant finding noted (Figure 4). 

Red blood cells

**Figure 5:**
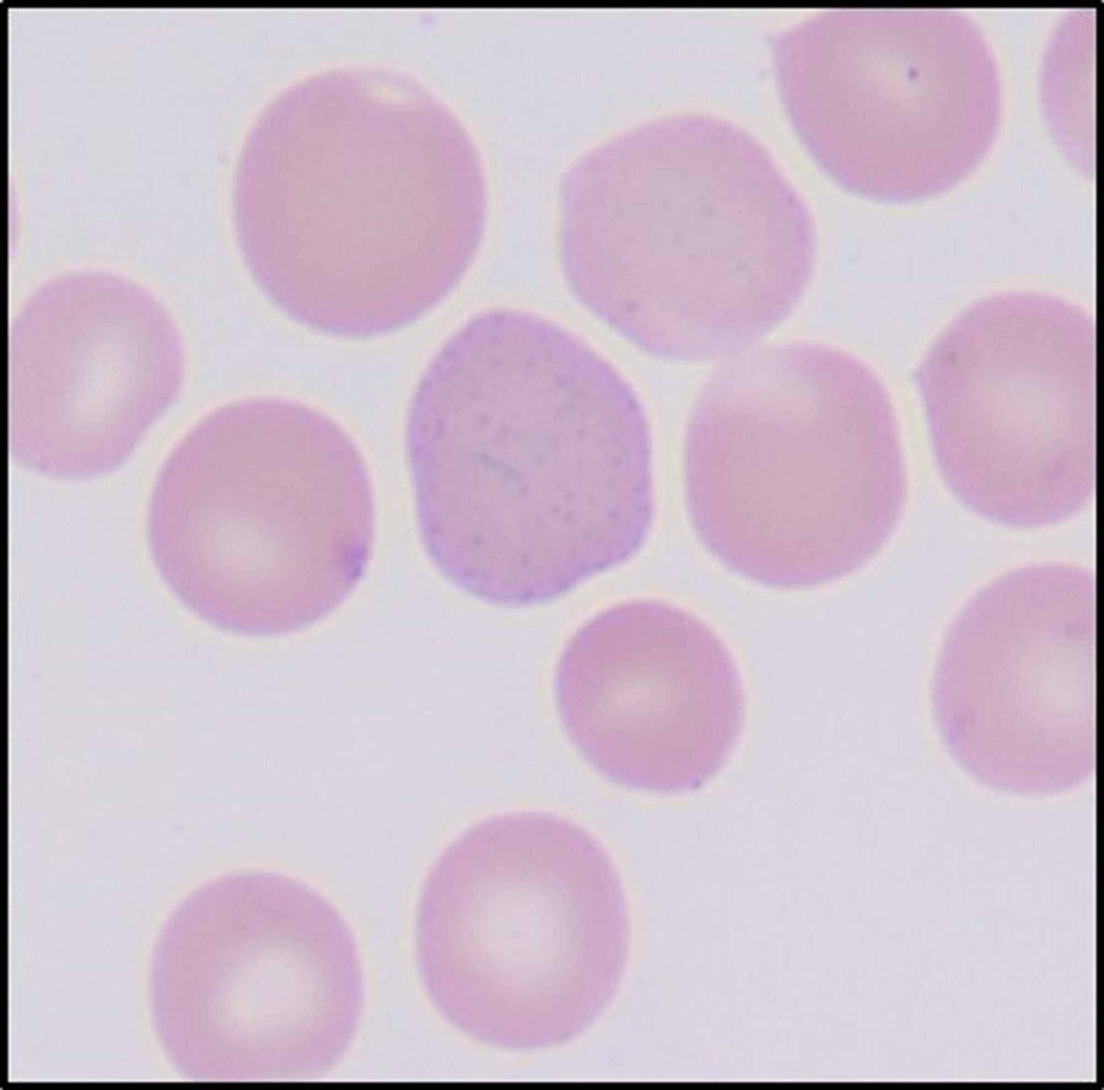
Red blood cell with basophilic stippling. (Original magnification ×1000 oil immersion.)

The red blood cells were normocytic and normochromic with few nucleated red blood cells and coarse basophilic stippling (Figure 5). 

## Discussion

The severe acute respiratory syndrome coronavirus 2 was first described in Wuhan, China, in December 2019; and on March 11, 2020, the World Health Organization declared the novel coronavirus outbreak a global pandemic [1,8]. SARS-CoV-2 belongs to a group of enveloped positive-sense single-stranded ribonucleic acid virus and the pathogenic mechanisms via which the virus causes disease in humans are still evolving [9,10]. So far, studies have shown that the virus invades host cells by attaching to ACE-2 via its spike surface glycoprotein S [11]. Invasion of host cells by the virus initiates a series of events that leads to tissue injury and destruction. Studies also report that the cytopathic effect of the virus stimulates the innate and adaptive immune system resulting in T-cell activation, cytokine release, monocyte activation, and B-cell mediated antibody production [5]. 

The main laboratory abnormalities noted in COVID-19 patients are anemia, leukopenia particularly lymphopenia, and leukocytosis including neutrophilia and monocytosis [6,7,12,13]. In our study, anemia was the most common finding, followed by neutrophilia, neutrophilic left shift, and lymphopenia. Several hypotheses exist for the cause of lymphopenia, including direct viral toxicity due to ACE-2 receptor expression and cytokine-induced lymphopenia [14]. Our study identified several morphologic changes in peripheral blood cells of COVID-19 patients. The neutrophils showed abnormal nuclear shapes such as fetus-shaped nuclei, pi-shaped nuclei, C and donut-shaped nuclei, and cells with aberrant nuclear projections. The pseudo-Pelger-Huet deformity was also identified. These morphologic changes have been reported in other studies. However, to our knowledge, few authors have fully described the morphologic abnormalities in all leukocytes of affected patients [12,15-19]. For instance, Zini et al. described similar changes in their study seen in neutrophils, lymphocytes, and platelets [15]. Singh et al. described similar features in neutrophils, lymphocytes, and monocytes in a case report. The author also suggested that activated monocytes may indicate an improvement in the patient’s clinical condition [16]. Zhang et al. described that monocytes express ACE-2 receptors and are directly affected by COVID-19 leading to monocytosis and the presence of large, atypical, vacuolated monocytes in circulation [12]. 

Infrequent abnormalities such as leukoerythroblastic reaction have also been described by Mitra et al. [17]. The leukoerythroblastic reaction is usually seen with bone marrow fibrosis including myelofibrosis, myeloproliferative disorders, and cancers with metastatic disease to the bone marrow [17]. Nazarullah et al also stressed morphologic findings seen in COVID-19 patients, especially acquired Pelger-Huet anomaly and plasmacytoid lymphocytes [18]. Pozdnyakova et al. have described that abnormal monocyte and lymphocyte morphology is associated with milder disease [19].

A finding unique to our study is the presence of smudged neutrophils and basophilic stippling in RBCs. Smudge cells are remnants of leukocytes, usually lymphocytes that are fragile and are destroyed/smudged in the physical process of making a smear. The significantly distorted shapes of neutrophils in COVID-19 patients along with toxic granulation and vacuolation may be causative of the fragility of neutrophils in these patients. However, the exact pathophysiology and mechanism of this process need to be studied.

## Conclusions

In conclusion, our study identifies and describes a summary of morphologic changes in the peripheral blood cells of COVID-19 patients. Although this is a pilot study with a small sample, it is a step towards understanding the hematological manifestations of COVID-19. Knowledge of these constellations of morphologic changes in peripheral blood, if substantiated with larger studies, may help physicians diagnose COVID-19 in the absence of a negative RT-PCR/antigen test. In addition, some authors have already suggested an association between morphological changes in CBC and disease progression/outcome. If validated by larger studies, serial CBC and blood smear review in hospitalized patients may become an essential tool to help clinicians assign patients to a higher risk category based on morphologic findings and take management decisions accordingly.
